# Supplementary material for: Roscovitine enhances the bactericidal activity of the airway surface liquid of the cystic fibrosis bronchial epithelium but does not protect against Pseudomonas aeruginosa infection
Source: PLoS One. 2025 May 5;20(5):e0321996. doi: 10.1371/journal.pone.0321996 (PMC12052092; doi:10.1371/journal.pone.0321996)
Supplement: Attachment — Submitted filename: PONE-D-24-24759R1_rebuttal letter.docx [file pone.0321996.s003.docx]

**Dear Dr Looi,**

**Thank you for considering our work for potential publication in PLOS ONE. We are grateful to the referee for his interest in our article, "Roscovitine does not protect cystic fibrosis bronchial epithelium against Pseudomonas aeruginosa infection". We have addressed all the issues raised by the reviewer and have completed the different sections. Below you will find our point-by-point reply to the reviewer's comments, and you can see the modifications highlighted in yellow in the "Revised Manuscript with Track Changes".**

Thank you for submitting your manuscript addressing the therapeutic potential of roscovitine against *Pseudomonas aeruginosa* in cystic fibrosis (CF). This work tackles an important area of CF research, particularly given the challenges posed by multidrug-resistant pathogens. Your use of a 3D model of fully differentiated F508del-CFTR human bronchial epithelium is a notable strength, providing a robust experimental platform. However, there are certain areas, indicated below, where the manuscript can be refined to improve clarity, accuracy, and alignment with the presented data.

1) Your data presentation is generally thorough, but incorporating more direct references to numerical findings and statistical significance within the main text would enhance clarity. For instance, the observed improvement in ASL bactericidal activity at 25 µM should be quantified and discussed in greater detail. Ensuring that all conclusions are explicitly tied to the data while avoiding speculative extrapolations will improve the manuscript’s rigor and focus.

**We have highlighted the results of the ASL bactericidal activity at 25 µM as requested in the Results part (now lines 227-228). As requested, we have also discussed in greater details how our study revealed a nuanced effect of roscovitine on ASL bactericidal activity and epithelial protection in pwCF (now lines 294-313).**

2) The manuscript would also benefit from a more explicit discussion of its limitations. The small sample size (n=6) and the in vitro nature of the experimental model limit the generalizability of the findings. Additionally, the absence of mechanistic insights into why roscovitine enhances ASL bactericidal activity at 25 µM but fails to protect epithelial integrity leaves unanswered questions. Addressing these issues in a dedicated section would provide important context for readers and help position your work within the broader research landscape.

**We have discussed in greater details (now lines 294-313) and nuanced more our results. We mentioned unanswered questions: (i) the mechanisms underlying the disconnect between improved ASL bactericidal activity and lack of epithelial protection, (ii) whether the effectiveness of roscovitine may vary depending on the infecting pathogen and (iii) a concentration-dependent effect of roscovitine.**

3) The conclusions currently overstate the findings, particularly the assertion that roscovitine does not protect CF bronchial epithelium against *P. aeruginosa*. While the data clearly demonstrate the lack of epithelial protection, they also reveal enhanced bactericidal activity of the airway surface liquid (ASL) at 25 µM. This is an important observation that should be reflected more prominently. A more balanced presentation of these results, emphasizing their complexity, would strengthen the overall narrative. Revisions to the abstract, results, and discussion to reflect this duality are recommended.

**The conclusion has been rephrased to point out that our study revealed a nuanced effect of roscovitine on ASL bactericidal activity and epithelial protection in pwCF (now lines 320-326). The abstract has been modified also (now lines 53-54). The title has been modified in lines of our nuanced findings.**

4) The figures, though informative, could be made more intuitive. Figures 1 and 3, in particular, would benefit from additional annotations or more detailed captions to improve their interpretability. This would allow readers to better grasp the key findings at a glance without needing to refer back to the main text.

**Results (now lines 210-211) of Figure 1, 2 and 3 and the captions (now lines 216-220, 237-238) were more detailed.**

Overall, this manuscript has potential but requires significant revision. Specifically, the conclusions should be rephrased to reflect the nuanced findings, limitations must be discussed more thoroughly, and the presentation of data should be refined for clarity and precision. By addressing these issues, the study will make a valuable contribution to CF research and meet the standards expected for publication in *PLOS One*.
