# Supplementary material for: Roscovitine enhances the bactericidal activity of the airway surface liquid of the cystic fibrosis bronchial epithelium but does not protect against Pseudomonas aeruginosa infection
Source: PLoS One. 2025 May 5;20(5):e0321996. doi: 10.1371/journal.pone.0321996 (PMC12052092; doi:10.1371/journal.pone.0321996)
Supplement: Attachment — Submitted filename: PONE-D-24-24759R2_rebuttal letter.docx [file pone.0321996.s004.docx]

**Dear Dr Mohapatra,**

**Thank you for considering our work for potential publication in PLOS ONE.**

**Firstly, we have edited "patients with CF" for "people with CF" throughout the manuscript.**

**Secondly, the scale bars on images have been modified to be larger.**

**Thirdly, as suggested by reviewer #2, given the literature about Roscovitine and Mycobacterium abscessus, we have added a sentence mentioning some potential limitations of incorporating Roscovitine into a regimen for chronic infection (line 313-316).**
